# Whole-Genome Sequencing of *Pantoea* sp. Strain RIT388, a Potential Oral Opportunistic Pathogen Isolated from a Chewing Stick (*Distemonanthus benthamianus*)

**DOI:** 10.1128/MRA.01468-19

**Published:** 2020-02-27

**Authors:** Han Ming Gan, Anutthaman Parthasarathy, Kurtis R. Henry, Michael A. Savka, Bolaji N. Thomas, André O. Hudson

**Affiliations:** a Centre for Integrative Ecology–School of Life and Environmental Sciences, Deakin University, Victoria, Australia; b Genomics Facility, Monash University, Selangor, Malaysia; c The Gosnell School of Life Sciences, Rochester Institute of Technology, Rochester, New York, USA; d College of Health Science and Technology, Rochester Institute of Technology, Rochester, New York, USA; University of Maryland School of Medicine

## Abstract

In this study, we report the isolation, identification, characterization, and whole-genome sequence of the endophyte Pantoea sp. strain RIT388, isolated from Distemonanthus benthamianus, a plant known for its antifungal and antibacterial properties that is commonly used for chewing sticks.

## ANNOUNCEMENT

The genus *Pantoea* is made up of Gram-negative bacteria within the Erwiniaceae family of enterobacteria and contains both free-living and host-associating species (1, 2). These bacteria form yellow mucoid colonies and associate with a variety of hosts, which include plants, insects, larger animals, and humans (1, 3). Some *Pantoea* species are well-known plant pathogens (4–6). Pantoea agglomerans has been isolated from patients with septic conditions, catheters, and trauma wounds, as well as from those with nosocomial infections (7, 8). Fatal outbreaks in neonates are known (9, 10). Apart from Pantoea agglomerans, Pantoea septica, Pantoea dispersa, and Pantoea latae, strains previously considered plant-associated or environmental isolates, Pantoea allii and Pantoea eucalypti are considered clinical specimens (11, 12).

**FIG 1:**
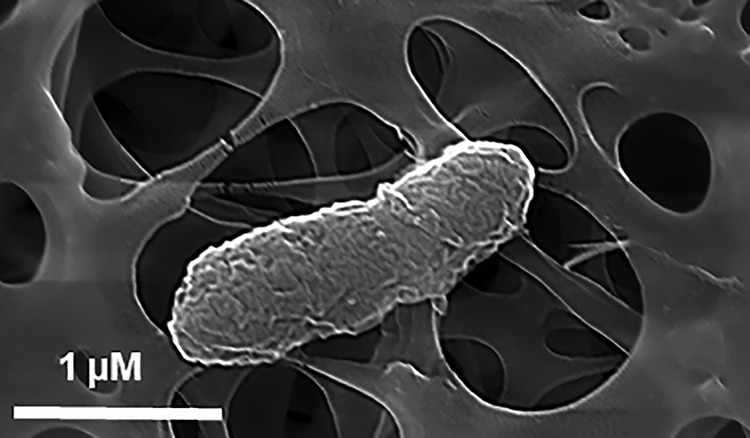
Scanning electron micrograph of *Pantoea* sp. RIT388, showing an elongated rod-shaped cell. Scanning voltage, 20 kV; magnification, ×74,100.

*Pantoea* sp. strain RIT388 was isolated on tryptic soy agar during a study to identify endophytic bacteria from Distemonanthus benthamianus (13). *Pantoea* sp. RIT388 cells are rods and are approximately 1.5 μm long (Fig. 1). D. benthamianus, used for oral hygiene, is a semideciduous perennial tree found in second-growth forests in Nigeria, Cameroon, and Ghana (14). A recent study showed that extracts from the bark possess bactericidal activity against Staphylococcus aureus and Streptococcus mutans, two bacterial species that are often associated with skin and dental infections, respectively (15).

Genomic DNA (gDNA) was isolated from a 5-ml culture of *Pantoea* sp. RIT388 grown in tryptic soy broth using the GenElute bacterial gDNA isolation kit (Sigma-Aldrich, USA) according to the manufacturer’s protocol. The gDNA was normalized to a concentration of 0.1 ng/μl based on a Qubit reading and processed with the Nextera XT library preparation kit (Illumina, San Diego, CA). The library was subsequently sequenced on a MiSeq sequencer located at the Monash University Malaysia Genomics Facility using a run configuration of 2 × 250 bp. Default parameters were used for all software unless otherwise noted. The raw sequencing reads were adapter trimmed and assembled *de novo* using Trimmomatic v0.39 (16) and Unicycler v0.4.7 (17), respectively. A total of 1,543,820 paired-end reads (∼387 Mb and 77× genome coverage) were generated and assembled into 68 contigs with a total length of 5,010,327 bp (GC content, 56.96%; *N*_50_ length, 221,699 bp). The assembled genome was then submitted to NCBI for annotation using the NCBI Prokaryotic Genome Annotation Pipeline (PGAP) v4.7 (18).

Some *Pantoea* species use quorum sensing via acyl homoserine lactone (AHL) signals to control gene expression based on cell density (19). However, despite harboring a *luxI* homolog (gene locus tag BBB56_18675), RIT388 does not accumulate AHL signals, as determined with a biosensor strain using the TraR receptor (20). This could mean that either a novel signal is produced or that the RIT388 *luxI* homolog is mutated. The latter has been shown in Vibrio fischeri, where mutations in *luxI* result in a nonfunctional protein (20).

### Data availability.

This whole-genome assembly of *Pantoea* sp. RIT388 has been deposited in GenBank under the accession number RMVG00000000 (assembly number GCF_003813865). Raw sequencing reads have been deposited in the SRA database under accession number SRR10522315 (BioProject number PRJNA327264).
